# External ventricular drain–related meningitis: prediction model or early diagnostic tool?

**DOI:** 10.1186/s40560-025-00842-7

**Published:** 2026-02-03

**Authors:** Muhammad Mohsin Sami, Ruqaiya Muhammad Naeem, Imran Zahid

**Affiliations:** 1 National Hospital and Cardiac Complex, Rahim Yar Khan, Punjab Pakistan; 2 Dr Faisal Masood Teaching Hospital, Sargodha, Punjab Pakistan

**Keywords:** External ventricular drain (EVD), Nosocomial meningitis, Prediction model

To the Editor,

We read with great interest the study by Raffenot et al. on risk factors for nosocomial meningitis (NM) in patients with external ventricular drains (EVDs) [1]. We commend the authors on their thorough and clinically impactful study. Their identification of factors such as cerebrospinal fluid (CSF) leakage and prolonged drainage offers valuable insight for daily practice.

The authors also develop a multivariable predictive model, incorporating CSF leakage, drainage duration, beta-lactam allergy, and a combined CSF variable (glucose ratio < 0.5 and protein > 1 g/L). This model demonstrates excellent discrimination (AUC 0.89) and the authors suggest that it could help identify high-risk patients earlier and guide closer monitoring and empirical therapy. We would, however, like to highlight one methodological point central to how the model’s performance and role should be understood.

The authors define NM using criteria adapted from Lozier et al., in which “probable” infection is based on specific cerebrospinal fluid (CSF) abnormalities: leukocyte count > 100/mm3, CSF-to-serum glucose ratio < 0.5, and protein concentration > 0.4 g/L [1, 2]. In the final model, very similar CSF parameters are then used as predictors, in particular a CSF-to-serum glucose ratio < 0.5 combined with protein > ^1 g/L.^

This introduces what is often called incorporation bias [3]. In effect, the model uses part of the definition of the outcome to “predict” the outcome, since the same CSF abnormalities used to define NM are also used as predictors. As a result, the model is largely identifying patients whose CSF profile already shows early signs of infection, rather than estimating who was at higher risk before these changes occurred. This distinction is important as it helps explain the model’s strong performance metrics (such as a high AUC and negative predictive value). These measures partly reflect how well the model recognizes patients whose CSF profile already meets (or nearly meets) the definition of NM. They do not reflect the ability to stratify risk at baseline, before those diagnostic changes appear. Clinically, the model may be more useful for early recognition in this retrospective study than for baseline risk prediction at EVD insertion.

We believe it is important for readers to recognize this distinction. Presenting the model in predictive terms may lead some readers to overestimate the extent to which it can inform early decision-making around NM. Although the authors acknowledge that the model is yet to be validated for risk prediction, misunderstanding of its intended use may have important clinical consequences. Clinicians may inappropriately start prophylactic antibiotics, increase CSF sampling frequency, or escalate monitoring unnecessarily based on CSF changes that indicate early infection rather than true risk. Misapplication can result in unnecessary treatments, increased antimicrobial resistance, greater burden of investigations, and poor resource allocation.

These methodological comments do not detract from the authors’ valuable contribution. Their identification of non-laboratory risk factors such as CSF leakage and prolonged drainage duration remains an important strength of the study. Moving forward, developing separate models for baseline risk prediction and early diagnostic support, clearly labeled and externally validated, would enhance clinical applicability and assist clinicians in both risk stratification and early diagnosis [4].

## Data Availability

No datasets were generated or analysed during the current study.

## Abbreviations

NM: Nosocomial meningitis
EVD: External ventricular drains
CSF: Cerebrospinal fluid
AUC: Area under the curve

## References

[1] Raffenot C, Doat-Sarfati V, Antonini F, et al. Risk factors for nosocomial meningitis in patients with external ventricular drainages. J Intensive Care. 2025;13:63. 41219997 10.1186/s40560-025-00828-5 PMC12604254

[2] Lozier AP, Sciacca RR, Romagnoli MF, Connolly ES. Ventriculostomy related infections: a critical review of the literature. Neurosurgery. 2002;51:170–82. 12182415 10.1097/00006123-200207000-00024

[3] Jong Y, Ramspek CL, Zoccali C, Jager KJ, Dekker FW, Diepen M. Appraising prediction research: a guide and meta-review on bias and applicability assessment using the prediction model risk of bias assessment tool (PROBAST). Nephrology. 2021;26(12):939–47. 34138495 10.1111/nep.13913 PMC9291738

[4] Collins GS, Dhiman P, Ma J, et al. Evaluation of clinical prediction models (part 1): from development to external validation. BMJ. 2024;384:e074819. 38191193 10.1136/bmj-2023-074819 PMC10772854

